# Isolation and Culture of Single Microbial Cells by Laser Ejection Sorting Technology

**DOI:** 10.1128/aem.01165-21

**Published:** 2022-02-08

**Authors:** Peng Liang, Bo Liu, Yun Wang, Kunxiang Liu, Yinping Zhao, Wei E. Huang, Bei Li

**Affiliations:** a State Key laboratory of Applied Optics, Changchun Institute of Optics, Fine Mechanics and Physics, Chinese Academy of Sciences, Changchun, People’s Republic of China; b University of Chinese Academy of Sciences, Beijing, People’s Republic of China; c Oxford Suzhou Centre for Advanced Research (OSCAR), University of Oxford, Jiangsu, People’s Republic of China; d Academy for engineering and technology, Fudan University, Shanghai, People’s Republic of China; e Department of Engineering Science, University of Oxford, Oxford, United Kingdom; Kyoto University

**Keywords:** single-cell isolating, single-cell culturing, laser-induced forward transfer, three-layer LIFT chip, fluorescence, single bacterial cell isolation and culture

## Abstract

Single-cell isolation and cultivation play an important role in studying physiology, gene expression, and functions of microorganisms. A series of single-cell isolation technologies have been developed, among which single-cell ejection technology is one of the most promising. Single-cell ejection technology has applied laser-induced forward transfer (LIFT) techniques to isolate bacteria, but the viability (or recovery rate) of cells after sorting has not been clarified in current research. In this work, to keep the cells alive as long as possible, we propose a three-layer LIFT system (top layer, 25-nm aluminum film; second layer, 3 μm agar media; third layer, liquid containing bacteria) for the isolation and cultivation of single Gram-negative (Escherichia coli), Gram-positive (Lactobacillus rhamnosus GG [LGG]), and eukaryotic (Saccharomyces cerevisiae) microorganisms. The experiment results showed that the average survival rates for ejected pure single cells were 63% for Saccharomyces cerevisiae, 22% for E. coli DH5α, and 74% for LGG. In addition, we successfully isolated and cultured the green fluorescent protein (GFP)-expressing E. coli JM109 from a mixture containing complex communities of soil bacteria by fluorescence signal. The average survival rate of E. coli JM109 was demonstrated to be 25.3%. In this study, the isolated and cultured single colonies were further confirmed by colony PCR and sequencing. Such precise sorting and cultivation techniques of live single microbial cells could be coupled with other microscopic approaches to isolate single microorganisms with specific functions, revealing their roles in the natural community.

**IMPORTANCE** We developed a laser-induced forward transfer (LIFT) technology to accurately isolate single live microbial cells. The cultivation recovery rates of the ejected single cells were 63% for Saccharomyces cerevisiae, 22% for E. coli DH5α, and 74% for Lactobacillus rhamnosus GG (LGG). With coupled LIFT with a fluorescence microscope, we demonstrated that single cells of GFP-expressing E. coli JM109 were sorted according to fluorescence signal from a complex community of soil bacteria and subsequently cultured with 25% cultivation recovery rate. This single-cell live sorting technology could isolate single microbes with specific functions, revealing their roles in the natural community.

## INTRODUCTION

Single-cell biotechnology is of great importance in the study of the growth, physiology, function, and biodiversity of microorganisms, especially for the as-yet-unculturable microorganisms in nature (1–4). Single-cell isolation techniques can play a vital role in the fields of single-cell genomics (5), neurobiology (6), and analysis of disease processes (7). To date, there are a number of single-cell-isolating methods available, such as manual micromanipulation, robotic micromanipulation (8–10), fluorescence-activated cell sorting (FACS) (11, 12), magnet-activated cell sorting (MACS) (13, 14), laser capture microdissection (LCM) (15), optical trapping (16, 17), and laser-induced forward transfer (LIFT) (18). Micromanipulation is a commonly used method in the laboratory. However, its throughput is relatively low and requires highly skilled professional training (19). FACS was introduced in 1969 by Leonard Herzenberg (20) and is of wide application with high throughput. FACS requires the samples to be resuspended in liquid solution (21, 22). Similar to FACS, MACS depends on a magnetic force to isolate cells from the cell suspension in a magnetic field (19). It is a challenge to use FACS and MACS to reveal spatial distribution of microbes and directly analyze complex samples *in situ*, such as soils, sludges, and sediments. LCM uses a focused laser to cut a cell from its surroundings, which is normally used for fixed tissue (15). Optical trapping directly captures the cell by optical forces (16). However, the throughput of optical tweezers is usually low (17).

LIFT is a promising method for precise single-cell isolation (18), which was exploited in 1986 when Bohandy et al. transferred copper (Cu) onto a silicon substrate using laser irradiation (23). Since then, LIFT has evolved and is widely used as a printing method which can transfer a great range of materials from electronics to cells and liquid to solid (24). The ejection mechanism of LIFT is dependent on thickness of the coating layer, material, and laser power and duration, but the basic setup is similar (24). When a laser pulse shines on the surface of the coating layer, it will absorb the laser energy and vaporize, and the material on it will be pushed away under the gas pressure (25–30) or shock wave (31, 32) induced by heating.

In the LIFT isolating process, we are able to observe the isolation of a single cell under the microscope, and LIFT can also be combined with other optical techniques such as fluorescence imaging (33) and Raman spectroscopy (25–27, 34, 35). LIFT has been applied for isolation and cultivation of microbial cells as well because of its ability to isolate the bacterial cells without destroying the microenvironment (for example, ejecting the bacteria and the surrounding soil together, but not focusing on single-cell ejection). Haider et al. used titanium oxide as the energy absorption layer and studied the effect of laser energy on the viability of yeast and Escherichia coli (36). Some researchers made use of LIFT’s advantage to isolate and culture the “unculturable” microorganisms from soil in nature (37) or analyze the soil microbial community (38). However, the initial challenge is the ability to isolate bacterial cells while maintaining their viability. Hence, the isolation of single live bacterial cells by LIFT and achieving subsequent cultivation is an important step toward dissecting complex microbial communities and the study of uncultured bacteria.

In this work, in order to improve the survival rate of separated cells, we developed a simple three-layer LIFT system to precisely isolate and culture single cells of typical yeast Saccharomyces cerevisiae, Gram-negative bacterium E. coli, and Gram-positive bacterium Lactobacillus rhamnosus GG (LGG) as a proof of concept. A microscopic imaging system was introduced during this work, which can visualize the location and ejection of LIFT-based single-cell sorting. The results showed that we successfully isolated and cultured bacterial or yeast cells at the single-cell level, which is not possible with traditional LIFT (Fig. S1 in the supplemental material). Furthermore, we demonstrated that green fluorescent protein (GFP)-expressing E. coli mixing with soil bacteria can be isolated and subsequently cultured using this system coupled with fluorescence detection.

## RESULTS

### Ejection and collection of single cells.

**FIG 1:**
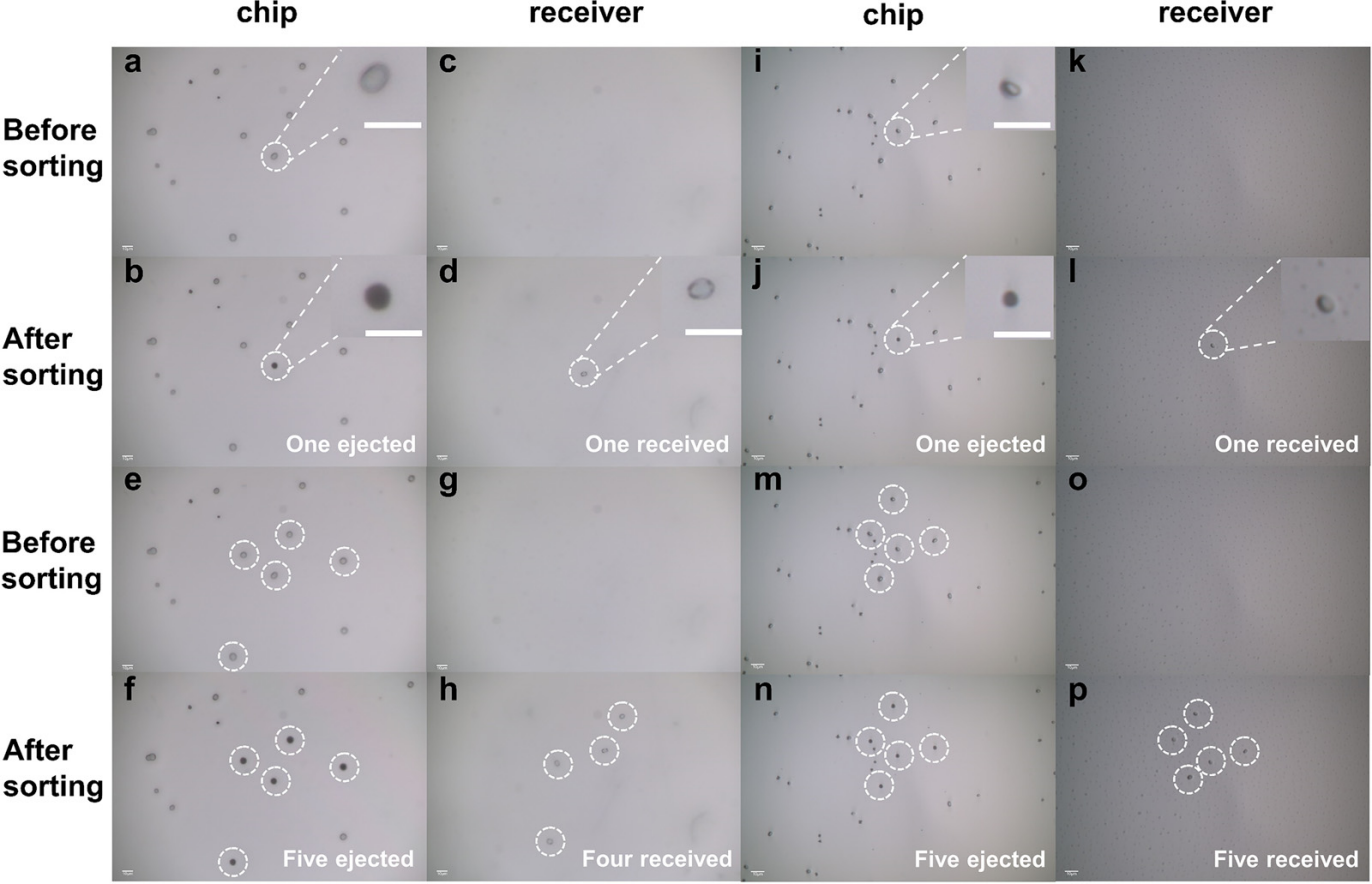
Isolating and receiving single S. cerevisiae and E. coli DH5α cells one by one; bar represents 10 μm. (a to d) Results of one single S. cerevisiae cell isolating and receiving. (a) Cell on chip before ejecting. (b) Cell was ejected, and a dark spot was left. (c) Blank on the receiver before receiving. (d) The ejected cell was received. (e to h) Results of isolating 5 S. cerevisiae cells and receiving. (e) Cells on chip before ejecting. (f) Five cells were ejected, and dark spots were left. (g) Blank on the receiver before receiving. (h) The ejected cells were received. Of note, only 4 S. cerevisiae cells were viewed in panel h. Actually, the fifth cell was also collected, but it was not in the same field of view as these four cells under the microscope. (i to l) Results of one single E. coli DH5α cell isolating and receiving. (i) Cell on chip before ejecting. (j) Cell was ejected, and a dark spot was left. (k) Blank on the receiver before receiving. (l) The ejected cell was received on the receiver. (m to p) Results of isolating 5 E. coli DH5α cells and receiving 5 E. coli DH5α cells. (m) Cells on chip before ejecting. (n) Five cells were ejected, and dark spots were left. (o) Blank on the receiver before receiving. (p) Five ejected cells were received.

Precise ejection and collection of single cells are important to single-cell isolating and culturing. In this work, Saccharomyces cerevisiae and E. coli were used to verify the ability of the three-layer LIFT system for isolation and capture of single live cells. To image the isolated cells, we used a 0.17-mm-thick transparent cover slide as the receiver to show that the cells were received (Fig. 1). The individual Saccharomyces cerevisiae and E. coli cells on the chip and the receiver could be clearly observed by adjusting the bottom objective to focus on different planes, and the distance between the chip and receiver was about 250 μm. As shown in Fig. 1, single Saccharomyces cerevisiae (Fig. 1a to d) and E. coli (Fig. 1i to l) cells were ejected from the chip, leaving a mark on the chip (no light reflected from the film into the camera because the aluminum film broke, so a dark spot was left), and the ejected cells were able to be retrieved on the receiver. When five single cells of Saccharomyces cerevisiae and E. coli were ejected, we could find five single cells on the receiver afterward. The distribution of the ejected cells on the receiver was similar to that originally on the chip (Fig. 1m to p). We received five single Saccharomyces cerevisiae cells on the receiver after ejecting five individual cells (Fig. 1e to h), but one of them was out of the viewing field (data not shown). These results suggested that the single-cell sorting system could precisely isolate individual yeast and bacterial cells.

### Sorting single cells into liquid medium for cultivation.

**FIG 2:**
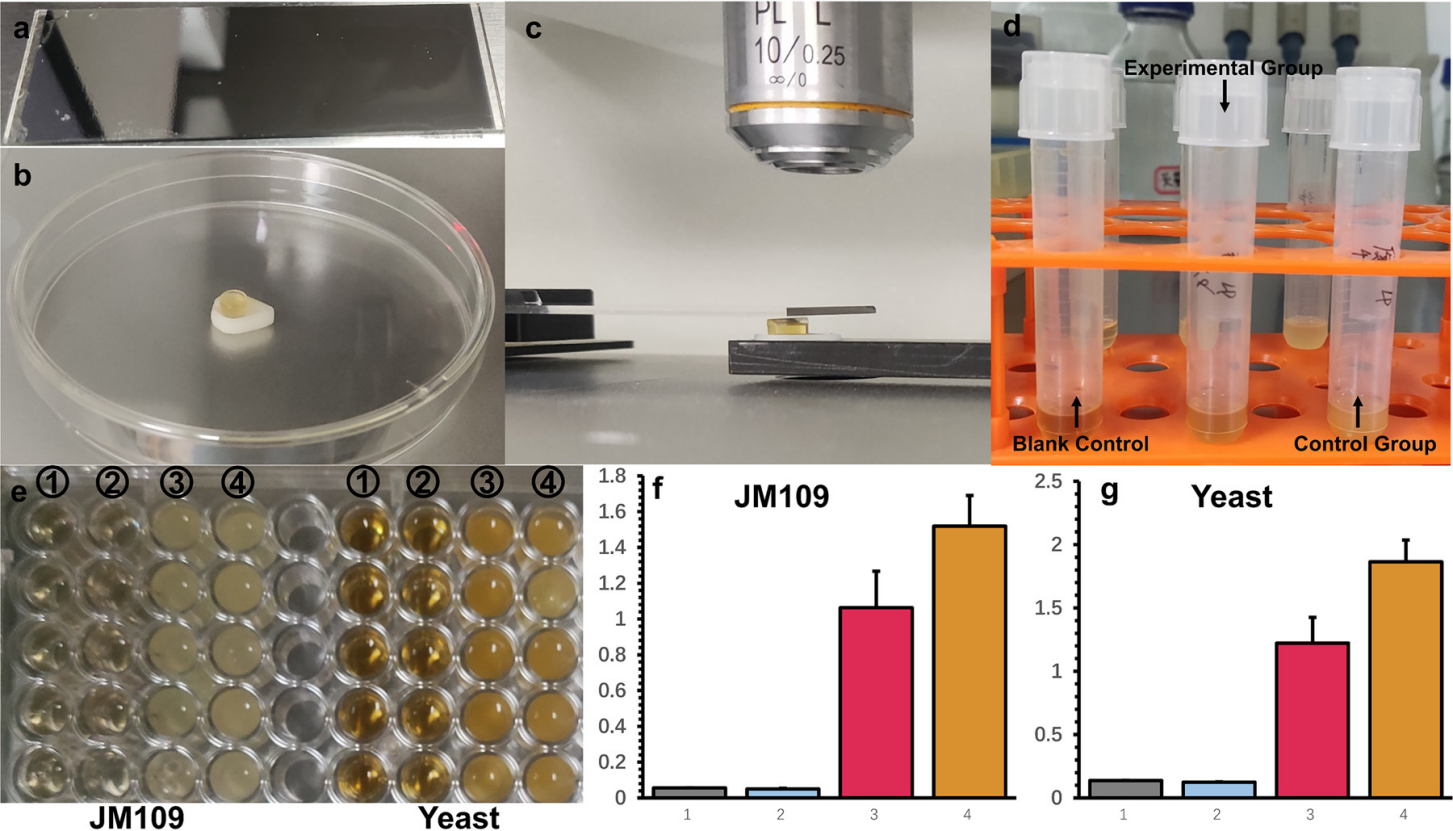
Ejection of a single cell into liquid medium for cultivation. (a) The LIFT chip coated with agar layer. (b) PCR tube cap with 40 μl liquid culture medium (40% D_2_O). (c) Ejection process. (d) Liquid culture medium was transferred into a tube containing 1 ml liquid culture medium (40% D_2_O) and cultured after 12 h. (e) Division into 5 groups for OD_600_ tests (Cytation 5; Biotek). (e1) Liquid culture medium (40% D_2_O) only; (e2) blank control, ejection within the cell-free area as negative controls; (e3) experiment group, with ejection 1 single bacterial cell into the cap; (e4) control group, bacteria without ejecting. (f) OD_600_ of JM109. (g) OD_600_ of yeast.

To verify the ability of this system of single-cell ejection and cultivation, we ejected single Saccharomyces cerevisiae or E. coli JM109 cells into 40 μl (40% D_2_O) yeast extract-peptone-dextrose (YPD) or LB broth, respectively (Fig. 2). Then, 40 μl liquid was transferred into a tube with 2 ml liquid medium and cultured for 48 h. We found the experimental group turn turbid, but the controls remained clear. We measured the optical density at 600 nm (OD_600_) with 5 biological replicates for both Saccharomyces cerevisiae and E. coli JM109, and the results are shown in Fig. 2f and g. The display of the C-D band (2,040 to 2,300 cm^−1^) of single-cell Raman spectra indicated that these cells were metabolically active (Fig. S7).

### Sorting single cells onto agar plates for cultivation.

**FIG 3:**
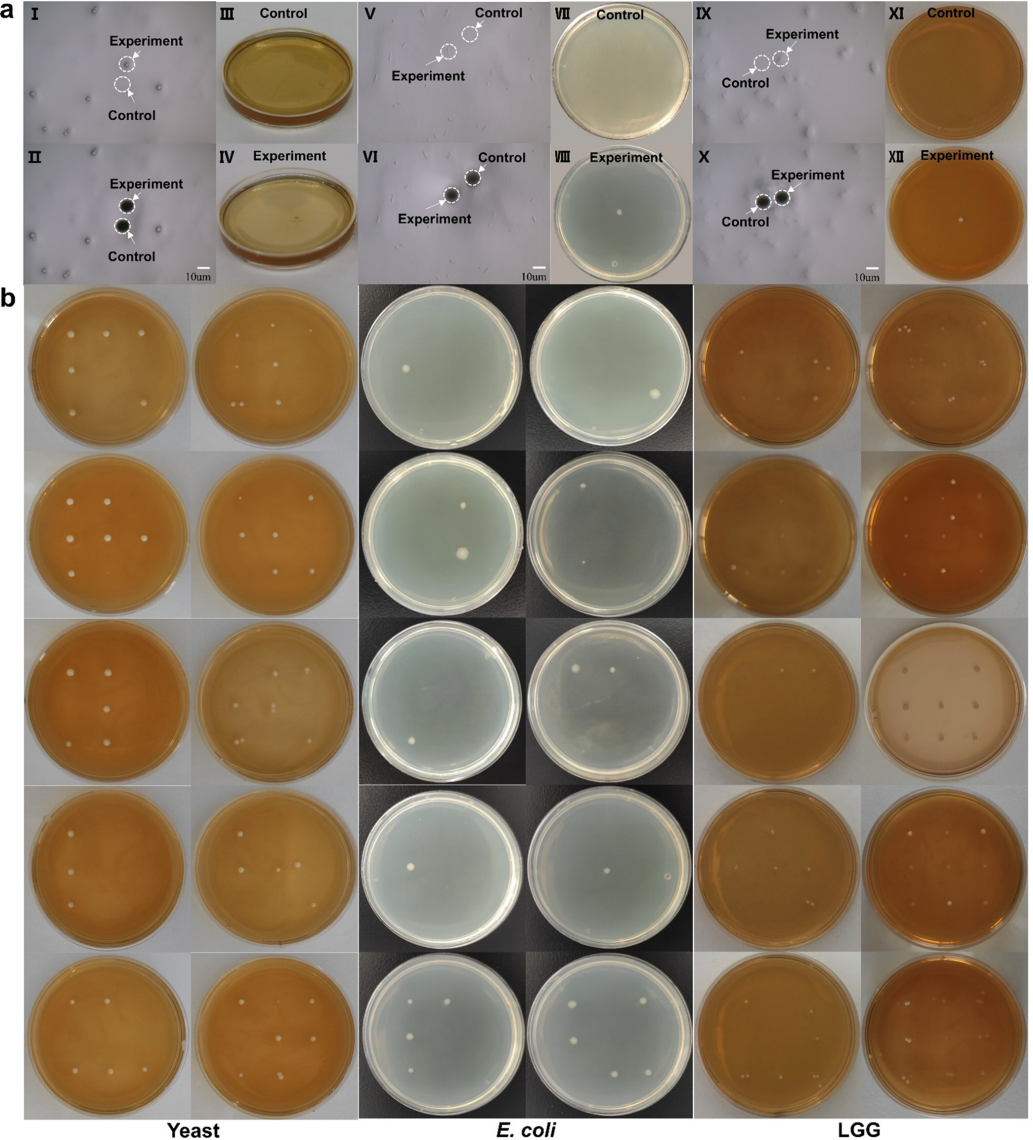
Ejecting single cells (S. cerevisiae, E. coli DH5α, and Lactobacillus rhamnosus GG) onto agar plates for cultivation. (a) The two black holes of panels II, VI, and X are left by the laser (no light reflected from the aluminum film into camera because the film was broken by the laser). We first ejected the “control black hole” around the cell into the petri dish and then ejected the target cell (corresponding to the experiment black hole) into another petri dish. No colony grew on the control petri dish, while a colony grew on the experiment petri dish, which shows that we can precisely transfer the target cell rather than eject the other around it. (aI to aIV) Results of sorting one single S. cerevisiae cell and culturing for 36 h. (aI) Chip before sorting. (aII) Chip after sorting. (aIII) No colony grew in the control group after culturing for 36 h. (aIV) A single colony grew in the experimental group after culturing for 36 h. (aV to aVIII) Results of sorting one single E. coli DH5α cell and culturing for 16 h. (aV) Chip before sorting. (aVI) Chip after sorting. (aVII) No colony grew in the control group after culturing for 16 h. (aVIII) A single colony grows in the experimental group after culturing for 16 h. (aIX to aXII) Results of sorting one single LGG cell and culturing for 48 h. (aIX) Chip before sorting. (aX) Chip after sorting. (aXI) No colony grew in the control group after culturing for 48 h. (aXII) A single colony grew in the experimental group after culturing for 48 h. Bar represents 10 μm. (b) Culturing results of isolated single E. coli DH5α, Lactobacillus rhamnosus GG (LGG), and S. cerevisiae yeast cells in each receiving position. The left two rows show ejection of one single E. coli cell into predetermined areas on the petri dish and culturing for 16 h, the middle two rows show ejection of one single LGG cell into predetermined areas on the petri dish and culturing for 48 h, and the right two rows show ejection one single S. cerevisiae yeast cell into predetermined areas on the petri dish and culturing for 36 h (sometimes two or more yeast or LGG cells are bonded together; the double or multiple colony that appears may be caused by ejection of these bonded yeasts or LGG).

Saccharomyces cerevisiae, E. coli DH5α, and Lactobacillus rhamnosus GG (LGG) were chosen as the representative eukaryotic and prokaryotic Gram-negative and Gram-positive cells for the single-cell sorting and cultivation in this study. The control group with the blank area near the targeted cells ejected did not lead to any colony growth on the receiving agar plates (Fig. 3a, agar plates in the first row). A single-cell sorting of Saccharomyces cerevisiae, E. coli, and LGG formed one single colony on the corresponding agar plates (Fig. 3a, agar plates in the second row), demonstrating that the sorted single cells after ejection were still alive and able to form single colonies. This showed that single-cell isolating and culturing could be indeed achieved using this three-layer LIFT system.

Figure 3b shows the results of single-cell ejection and cultivation of E. coli DH5α, LGG, and Saccharomyces cerevisiae at 9 different predetermined receiving places in one petri dish, respectively. Ten replicates were performed in each case, and the average recovery rates of single E. coli DH5α, LGG, and Saccharomyces cerevisiae cell ejection were 22%, 74%, and 63%, respectively (Fig. 3b and Table S2). It is likely that Gram-positive LGG was robust to survive after ejection sorting due to its thick cell wall protection.

To verify the sorted single cells were the originally targeted cells, five colonies of each species were randomly selected for colony PCR using yeast 18S (for Saccharomyces cerevisiae) and universal 16S rRNA primers (E. coli DH5α and LGG), respectively (Fig. S3). The PCR products were purified and sequenced. The sequencing results confirmed that the sorted cells were the original target cells. The quality of the Sanger sequencing results was good and clear without ambiguous reading.

The results demonstrate that single yeast and bacterial cells can be sorted by the LIFT ejection system while remaining alive to be able to form colonies.

### Sorting GFP-expressing cells for cultivation from the soil microbial community.

**FIG 4:**
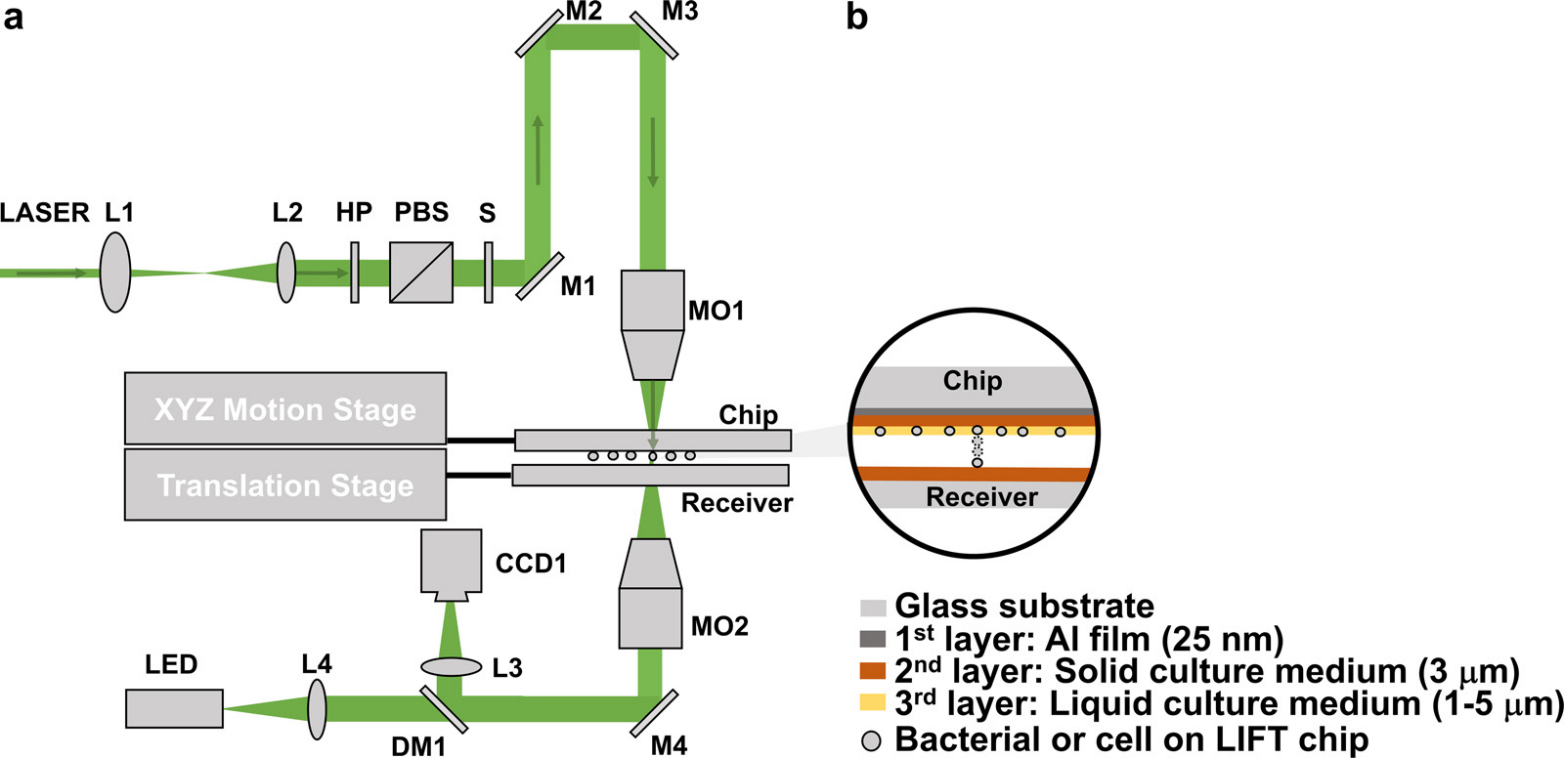
(a) Schematic of the laser induced forward transfer (LIFT) system used for single-microorganism isolating and culturing. L1 to L4, lenses; HP, half-waveplate; PBS, polarizing beam splitter; S, Shutter; M1 to M4, mirrors; DM1, dichroic mirror; MO1 to MO2, microscopy objectives. (b) Three-layer structure. 1st layer, Al film; 2nd layer, solid culture medium; 3rd layer, liquid culture medium.

By replacing the DM1 (Fig. 4) with a fluorescence cube (MDF-GFP2; Thorlabs), the system could distinguish cells expressing fluorescent protein. To prove the basic process, we identified green fluorescent protein-expressing E. coli JM109 (pGFP) from its mixture with E. coli DH5α (Fig. S4), sorted single cells of E. coli JM109 (pGFP), and then cultivated them on an LB agar plate (Fig. S5). From the mixture of E. coli JM109 (pGFP) and DH5α, we sorted 9 single cells in each petri dish and 162 single cells on 18 petri dishes. We have managed to culture 41 colonies for 162 attempts (Fig. S5). The cultivation recovery rate of single-cell sorting was 25.3% (41/162).

**FIG 5:**
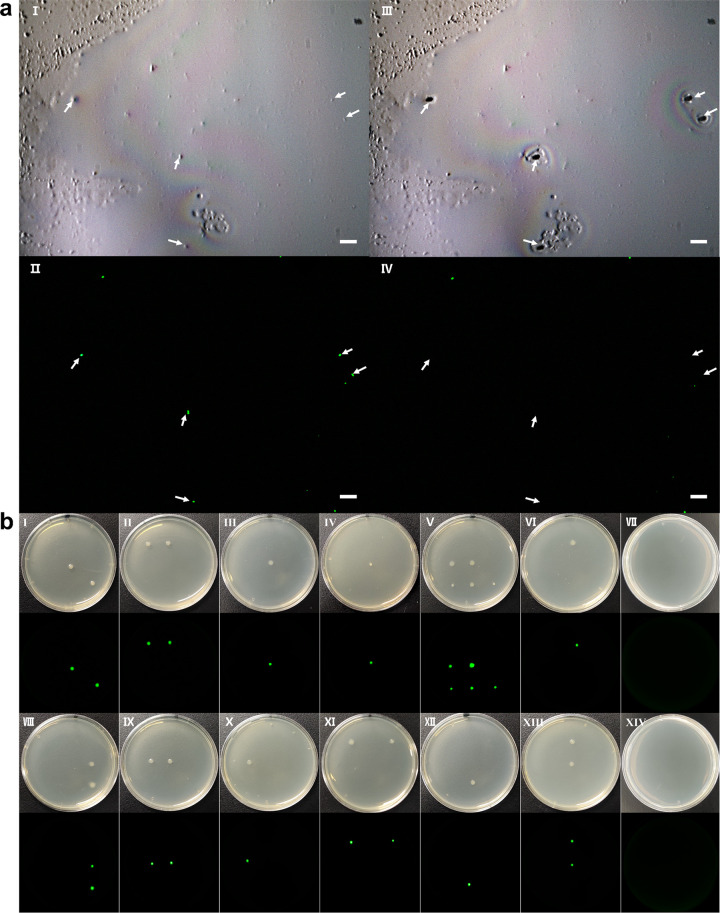
Fluorescence isolation of single JM109 cells from soil sample. (aI) Image before ejection. (Dots show in this picture but do not show green, and they represent the microbials or impurities in the soil.) (aII) Fluorescence image before ejecting. (Green dots represent JM109 cells). (aIII) Image after ejecting. (a, IV) Fluorescence image after ejecting. Bar represents 10 μm. (b) Culturing results of isolated single JM109 cells (pGFP) from soil samples, cultured after 24 h. The black picture below each panel is the corresponding fluorescence picture, taken by the ChemiDoc MP imaging system (Bio-Rad) operating on DyLight 488 mode (excitation 488 nm and emission 532 nm). (bI to bVI) First experiment, where we ejected 9 single cells in each petri dish and 81 single cells on 9 petri dishes. In total, 12 colonies grow on 6 petri dishes. (bVIII to bXIII) Second experiment, where we ejected 9 single cells in each petri dish and 81 single cells on 9 petri dishes. In total, 10 colonies grew on 6 petri dishes. The single cell’s recultivation ratio was about 13.6% (22/162). (bVII) Control group, which was placed in the air. (bXIV) Control group, which was ejecting the blank place around the cell and receiving.

To demonstrate the ability of the sorting system to specifically target bacteria from the complicated microbial community, we selectively isolated E. coli JM109 (pGFP) from its mixture with a soil microbial community (Fig. 5). Fluorescence imaging identified and sorted five single cells of GFP-expressing E. coli JM109 (pGFP) among the soil bacterial community (Fig. 5a, panels I and II). Fluorescent imaging after the sorting shows that these five cells disappeared (Fig. 5a, panels III and IV). The fluorescent cells were ejected one by one onto petri dishes to culture (Fig. 5b). The cultivation recovery rate of single cells of E. coli JM109 (pGFP) sorting from the soil microbial community was about 13.6% (22/162). All sorted and recultivated cells showing GFP and the control group (Fig. 5b, panel VII, which was placed in the air; Fig. 5b, panel XIV, which performed ejection within the cell-free area as negative blank controls) have no cell growth. Colony PCR for the GFP gene and subsequent sequencing confirm that those GFP cells were E. coli JM109 (pGFP) (Fig. S6). We also checked the isolated colonies under a fluorescence microscope, and all cells showed GFP. The results confirmed that the isolated cells were the targeted E. coli JM109 with pGFP.

## DISCUSSION

### Precise single-cell ejecting and capturing.

In this study, single-cell fluorescence sorting (Fig. S4 in the supplemental material; Fig. 5), capturing (Fig. 1), and culturing (Fig. 2, 3, and 5; Fig. S5) have been accomplished by using a three-layer LIFT system. Although LIFT has been reported in printing a great range of materials from electronics to cells and liquid to solid, it has not been easily applied in accurate single-cell ejecting and culturing because of the heat and force in the LIFT process. Much research focused on printing mammal cells from liquid layer (33, 39–41) with a high-power or shorter-duration laser (compared to this work), but a microbial cell is smaller than a mammal cell. We need the liquid layer to be thin enough (thinner than/equal to the diameter of the Saccharomyces cerevisiae or E. coli) so that the Saccharomyces cerevisiae or E. coli cells cannot move freely in the liquid layer and we are able to focus on the target cell to eject.

When doing experiments, the environmental conditions such as the platform vibration, airflow, or other microenvironments could affect the success rate of cell ejecting, receiving, and culturing. These factors can mostly be avoided by placing the experiment setup on an active-vibration isolation optical table in a confined environment. In addition, the laser spot’s position and energy also need to be carefully adjusted. If the spot deviates too far from the target or the energy is not moderate, three cases may happen. First, the cell may not be successfully ejected (Fig. S8b, panel I). Second, the ejected cell flies away and does not land on the right receiving position. A fast movie shows that the cell rebounded when touching the receiver (cover glass) and then fell back (Fig. S9 and Video S1 [supplemental file 2]). Finally, other cells around the target cell were also ejected, which isolated unwanted cells and caused contamination. To address the issues listed above, we use a 500-nJ laser pulse to eject the Saccharomyces cerevisiae and 300 nJ for E. coli and Lactobacillus rhamnosus GG (LGG). The effect of the energy from the laser pulse, the agar’s thickness, and other materials will be studied further in the future.

As for the physical mechanism of ejection, it depends on a few parameters, such as laser pulse energy, laser pulse duration, and thickness of the film. In previous reports, it was found that the shock wave produced the force to push the cells when the laser pulse duration was at the level of picosecond or femtosecond (31, 32). When the pulse duration was at the level of nanosecond or longer, the gas pressure was dominated (25–30). Since our pulsed laser is at nanoseconds, we tend to think the force of the isolation is the shock wave of deformed agar film pushed by the gas pressure from vaporization of the aluminum film (Fig. S8b, panels I to III). Further investigation in the future is required to understand the mechanism of the ejection sorting force.

### Single-cell viability and cultivability.

Laser radiation, heat, and force in ejecting, flying, and landing processes and a dry environment all may cause damage to single cells in the LIFT process. This three-layer design could reduce the damage in some aspects and also cause some limitations. First, for the laser radiation, only a tiny amount (1 to 3%) of the laser pulse could pass through the 25-nm aluminum film (Fig. S8a) (tested by energy meter Vega [Ophir Photonics, Israel]); the laser pulse energy we use is tiny (500 nJ), so only 5 nJ (1% is 5 nJ) energy could pass through the aluminum film, and we consider that other reasons, such as the agar’s absorption and scatter, the laser energy absorbed by the cell, could be ignored. Also, the laser pulse duration is short (5 ns), unlike optical tweezers, which are more gentle, but individual cells are handled for larger periods of time, so the total energy deposited on a cell can be high and therefore damaging.

Second, regarding heat and force in the ejection, simulation results show that the ejection happened (about 10 ns after laser pulse) before the heat transferred to the cell (about 30 ns after laser pulse) so that the transferred cell avoided the heat damage. Compared with heat, force is difficult to be simulated, but as the intermediate layer, agar could reduce the pushing force to some degree.

The third limitation is flying and landing processes. These aspects were not well studied in this work, but from a video of the flying and landing (Fig. S10 and Video S2 [supplemental file 3], taken from the side view by a high-speed camera at 11,010-fps frame rate; PCO.dimax HS4; Germany), the velocity could be calculated at about 0.35m/s.

In the experiments, we found that maintaining moisture of bacterial cells is a key factor for isolating live single bacterial cells. On one hand, a dry environment may dehydrate bacteria, and some of the cells may die (Fig. S11); on the other hand, water around the cells protected from the heat causes damage in the LIFT process. Although some glycerol was added (8%) to slow down the liquid’s drying speed, it would still dry out after a long time (about 30 min) plating onto the chip. The ejection sorting can be potentially high throughput, as the ejecting time is nanosecond level. The limiting step is the process of receiving cells. In the future, a new membrane is needed to further slow down the liquid’s drying speed to keep the cell’s viability, and a new design of receiver will be employed to improve the throughput.

Although other adiabatic materials such as polyimide have been used as the intermediate layer, agar medium is still a good choice because it is very inexpensive and easy to obtain. Furthermore, agar is not as strong as polyimide, so it needs less energy to achieve single-cell transfer, especially for a microbial cell. We used only a 500-nJ laser pulse for Saccharomyces cerevisiae and 300 nJ for E. coli and LGG in the experiment.

This study accomplishes one single bacterial or yeast cell isolation and culturing using the LIFT technique. Armed with the live-cell sorting technology, sequencing of the targeted microbes becomes very easy, as we can obtain a large amount of genomic DNA from the cultured microbial cells. Combined with fluorescence imaging, the bacteria with fluorescence could be isolated from the sample and then cultured. This live sorting of single bacteria can be explored to combine with fluorescent and Raman spectroscopy to precisely isolate the microbes at the single-cell level.

## MATERIALS AND METHODS

### Apparatus setup for single-cell sorting.

The schematic of the experimental setup is illustrated in Fig. 4a. A 532-nm laser pulse with 5-ns full-width half-maximum (FWHM) duration was utilized for single-cell ejection. The laser beam was coupled into a 10× microscope objective (MO1) and focused on the aluminum film (25 nm) coated on the glass. To control the laser pulse, we designed a laser beam expander, a laser energy-adjusting module, and a shutter in the optical path (Fig. 4a). The laser beam expander (L1, focal length [*f*] = 15 mm; L2, *f* = 50 mm) was used to expand the laser beam from 1 mm to 3.3 mm, which can fit the 10× microscope objective. The laser energy-adjusting module contained a half-wave plate (HP) and a polarizing beam splitter (PBS); by adjusting the angle of the half-wave plate (HP), the laser pulse energy could be changed from 80 nJ to 1,300 nJ. The shutter (S) and laser were programmed to form a single laser pulse. The mirrors (M1 to M4) in the setup were used to change the direction of the laser.

The cell images were obtained by the bottom imaging system, and the imaging microscope objective (MO2) used here was a 50× Nikon objective. Both the LIFT chip and the receiver were mounted on a translation stage. When cells on the LIFT chip were being examined, the receiver was motored outside the light path. After a cell was targeted, the receiver would move to the right place to collect the ejected cell.

### LIFT chip and receiver.

For the LIFT chip, we proposed a three-layer design. A 25-nm-thick aluminum film was used as the first layer and coated on a glass slide. The aluminum film absorbs the laser pulse and will be heated and form the ejecting force. Then an agar medium (YPD agar for Saccharomyces cerevisiae, MRS agar for LGG, and LB agar for E. coli) film was spread as the second layer by a spreading machine. To make the solid culture medium film uniform, three phytic acid (PA)-Al^3+^ films were attached onto the surface of the aluminum film to make it hydrophilic (42). The procedure was performed as follows: the cleaned chips were soaked in 0.255 mmol/L PA solution for 10 min and then soaked in 55.5 mmol/L AlCl_3_ solution for 2 min, rinsed with ultrapure water, and dried with a rubber suction bulb. This process was repeated 3 times to obtain 3 layers of PA-Al^3+^ complex on the chip. Then, we heated the solid culture medium to a liquid state in an oven and spread it onto the surface of the chip with a spreading machine (KW-4A; Institute of Microelectronics of the Chinese Academy of Sciences) rotating at a speed of 500 rpm for 16 s and 1,000 rpm for 2 s. The thickness of the solid culture medium is roughly estimated as 3 μm under a microscope. After the LIFT chip was cooled in a 4°C refrigerator for a while, we directly spread the yeast cell solution or bacteria solution (the third layer) on the LIFT chip and then clamped it by a holder, which was mounted on an XYZ motion stage for further observation and selection under a microscope.

For the receiver, a PCR tube cap with 40 μl liquid culture medium and a 35-mm petri dish (Thermo Fisher) with agar medium were employed for cultivation in liquid environment and agar medium, respectively. Both kinds of receiver were fixed on a translation stage, and the distance between the receiver and the donor was about 2 mm. For the liquid culture medium, 40% D_2_O (vol/vol) was added to culture to the received cells for the Raman microspectroscopy. In addition, for the 35-mm petri dish receiver, we wrote a program to control the translation stage to stop at 9 different points corresponding to the 9 receiving points on the petri dish.

### Temperature control of the three-layer LIFT system.

The chip designed in this research contained three layers (Fig. 4b), and to evaluate this three-layer LIFT system’s ability to protect cells from heating damage, we developed a heat transfer model (Fig. S2a in the supplemental material) using the software package COMSOL. In this model, the first layer is aluminum film (25 nm thick), the second layer is agar (about 3 μm thick; this layer is solid culture medium, and its properties are similar to agar), and the third layer is water (3 μm thick; in fact, this layer is the liquid culture medium, and its properties could be thought of as water). The governing equation is a time-dependent heat transfer equation (equations 1 and 2). The laser pulse can be considered a heat source (equation 3). It has a Gaussian distribution in r (radial) direction as written in equation 4, and the energy variation along with time is written in equation 5.

(1) $$\text{ρ}C_{p}\frac{\partial T}{\partial t}+\text{ρ}C_{p}u\cdot \nabla T+\nabla \cdot q=Q$$

(2) q=−k ∇T

(3) $$Q=\left(1-R\right)\cdot \left(\frac{2\cdot E_{p}}{\text{π}\cdot r_{a}^{2}\cdot \tau }\cdot 1.133\right)\cdot f_{1}\left(r\right)\cdot f_{2}\left(t\right)$$

(4) $$f_{1}\left(r\right)=e^{\left[-2\cdot {\left(\frac{r}{r_{a}}\right)}^{2}\right]}$$

(5) $$f_{2}\left(t\right)=e^{\left[-2\cdot {\left(\frac{t-2\text{τ}}{\tau }\right)}^{2}\right]}$$

where ρ is density (kg/m^3^), *C_p_* is heat capacity (J/kg·K), *T* is temperature field (K), *t* is time (s), *u* is the velocity field (m/s), *q* is heat flux on the coating material (W/m^2^), *Q* is heat flux transferred from the laser (W/m^2^), *k* is thermal conductivity (W/m·K), *R* is aluminum film reflectance, *E_p_* is laser pulse energy (J), *r_a_* is beam radius (m), and τ is laser pulse width (s); 1.133 in equation 3 is the energy coefficient.

The physical parameters of each material are listed in Table S1. The density, thermal conductivity, and heat capacity are standard properties for aluminum and water. For agar, the density and thermal conductivity are calculated according to a previous report (43), and the heat capacity is the same as water. The initial temperature is set as the ambient temperature, 20°C. A few simplifications and assumptions are introduced to the computation process. First, there is no phase change in any materials. Second, deformation of the solid culture medium is considered minimal. Third, the heat can conduct freely between layers ignoring the thermal contact resistance. All these assumptions are made to simplify the simulation and to help the prediction of thermal effect on cells. The simulation results (Fig. S2c) suggest that the temperature of the third layer starts rising at about 30 ns. The laser pulse is about 10 ns, and the ejecting process happens at the moment the aluminum’s temperature reaches the evaporating point, which will complete in less than 10 ns. Therefore, theoretically, the cell should have been ejected before the heat was transferred to it. Hence, a cell ejected under this designed system can remain alive without heat damage.

### Cell cultivation, laser-induced transfer of a single cell, and recovery of sorted cells.

Single colonies from Saccharomyces cerevisiae, E. coli DH5α, E. coli JM109 (with a plasmid pGFP encoding a constitutive expression of GFP), and Lactobacillus rhamnosus GG (LGG) were inoculated into 2 ml YPD, LB, and LB with 100 μg/ml ampicillin and MRS broth, respectively. The yeast was cultured at 30°C, and E. coli DH5α was cultured at 37°C; both of them were cultured for 16 h with a shaking rate of 200 rpm to reach OD_600_ of 1.5, and LGG was cultured at 37°C without shaking.

For the LIFT process of single-cell ejecting and culture, the following different laser energies were applied for the three species: 300 nJ for E. coli and LGG and 500 nJ for Saccharomyces cerevisiae yeast. By controlling the upper XY motorized stage (Fig. 4a), we selected the target cell and made it under the laser pulse position, and then the receiver reached under the target cell to receive. After ejection, the receiver was moved out of the optical path. To avoid contamination, the experiment setup and the chip and receiver were all put into a laminar flow cabinet, which was exposed to UV light for 20 min before doing the experiment.

Single cells from LIFT isolation were put into 40 μl liquid culture medium or agar petri dishes for cultivation. Both liquid cultures and the receiving petri dishes were incubated at 30°C for 36 h for Saccharomyces cerevisiae, 37°C for 16 h for E. coli, and at 37°C for 48 h for LGG.

### Single-cell Raman spectra measurement.

To check the metabolic activity of the cultured cells, 40 μl liquid culture medium was transferred into a tube with 1 ml liquid culture medium containing 40% D_2_O. After cultivation, cells were harvested by centrifugation at 4,000 rpm for 5 min to remove supernatants. Then, the cells were collected and washed with deionized water three times. A single-cell Raman spectrometer (Preci SCS; Hooke Instruments Ltd., China) was employed to detect the Raman spectra of the single cells. The operating power of the laser was 3.5 mW, and the acquisition time was 5 s.

### Soil sample collection and treatment.

A soil sample (5 g) from a garden was collected and diluted with 50 ml deionized water, and then the samples were centrifuged with different speeds of 500, 1,000, 2,000, and 3,000 rpm, each for 5 min, to remove the debris. Then, the supernatant of the soil sample was harvested. The soil microbial community in 1 ml of the soil supernatant was harvested by centrifugation at 8,000 rpm for 5 min. Removing the supernatant, the pellet of the soil microbial community was resuspended and mixed with 1 ml E. coli JM109 with pGFP (∼10^8^ cells/ml).

### Colony PCR and sequencing.

For Saccharomyces cerevisiae, 5 colonies from the cultivation plate were randomly picked and mixed into 500 μl ultrapure water. Then, 2 μl of this solution, 10 μl *Taq*, 1 μl NL1 primer (10 μM concentration), 1 μl NL4 primer (10 μM concentration) (44, 45), and 6 μl diethyl pyrocarbonate (DEPC)-treated water (catalog no. R1600; Solarbio) were mixed together (20 μl) into a PCR tube. For E. coli and LGG, 5 colonies from the cultivation plate were randomly picked and mixed into 1 ml ultrapure water, and then 2 μl of the solution, 10 μl *Taq*, 1 μl 27F primer (10 μM concentration), 1 μl 1492R primer (10 μM concentration) (44), and 6 μl DEPC-treated water (R1600; Solarbio) were mixed together as the PCRs (20 μl).

The PCR was performed on a T100 thermal cycler (Bio-Rad). The amplification program used here was as follows: 95°C for 3 min and 35 cycles of 95°C for 15 s, 55°C for 30 s, and 72°C for 1 min; and 72°C for 5 min. The gel electrophoresis experiment was performed on PowerPac (Bio-Rad) at 130 V for 25 min, the amplified product fragments were sequenced, and sequencing was conducted by Sangon Biotech (ABI Prism 377XL). The 16S rRNA gene sequence of the sample was compared in the GenBank database, and the strain identification result was finally obtained.
