# Supplementary material for: Isolation and Culture of Single Microbial Cells by Laser Ejection Sorting Technology
Source: Appl Environ Microbiol. 2022 Feb 8;88(3):e01165-21. doi: 10.1128/aem.01165-21 (PMC8824209; doi:10.1128/aem.01165-21)
Supplement: Supplemental file 1 — Fig. S1 to S11, Tables S1 and S2. Download aem.01165-21-s0001.pdf, PDF file, 1.0 MB [file aem.01165-21-s0001.pdf]

Supplementary information

Isolating and culturing of single cells by laser ejection sorting technology

Peng Liang[a,b], Bo Liu[a,b] , Yun Wang[c], Kunxiang Liu[a,b] , Yinping Zhao[d], Wei E. Huang[c,e,*], Bei Li[a,f,*]

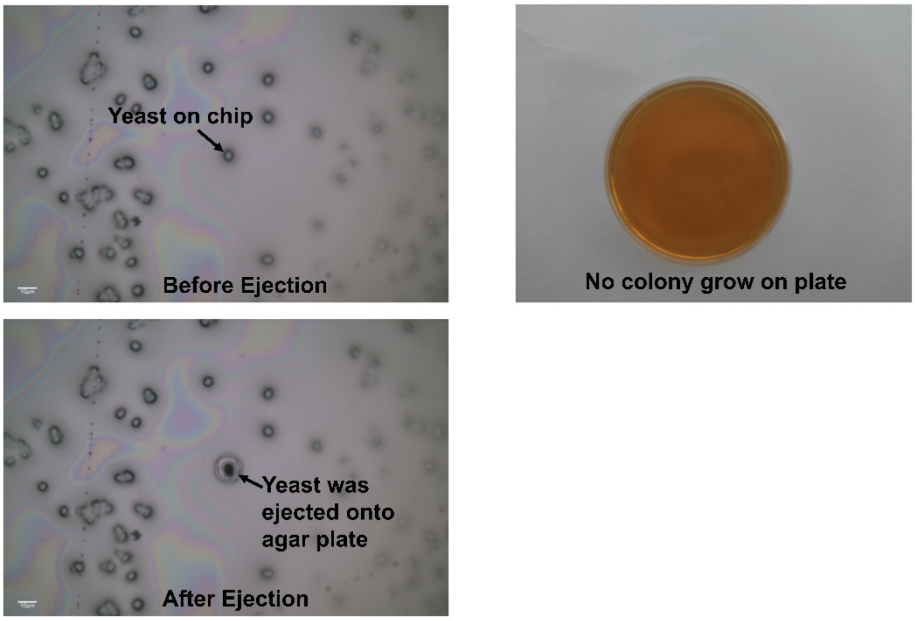

Supplementary Figure S1. Simple ejection of the cell cannot get cultivable colony.

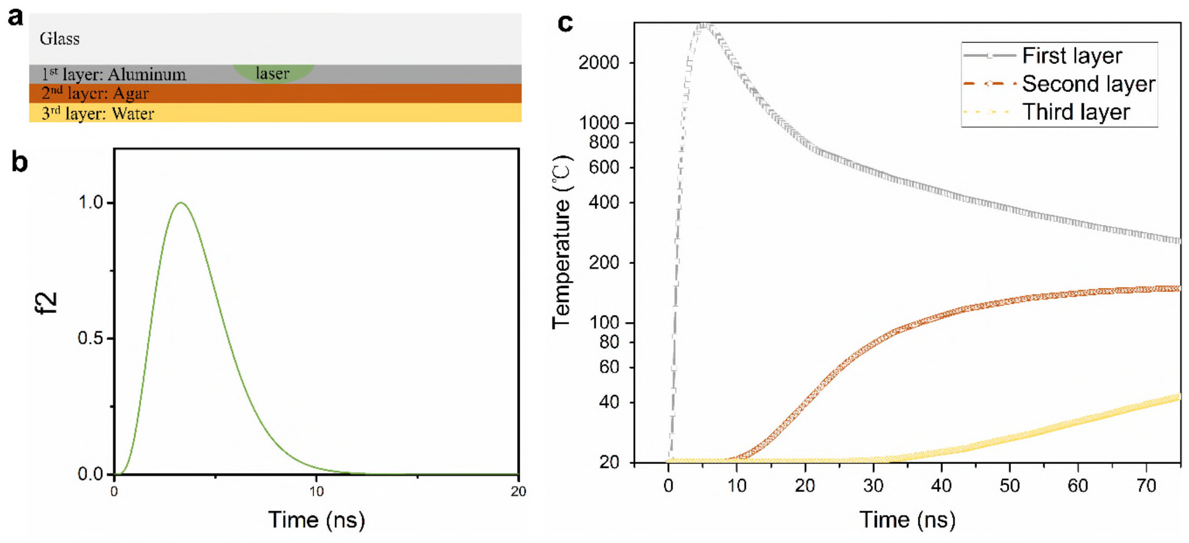

Supplementary Figure S2. Numerical simulation of the temperature on the surface of each layer with Comsol®. (a): Schematic of the FE model. (b): Laser pulse's energy variation along time. The single laser pulse ends at about 10 ns. (c): Temperature variation of each layer along time.

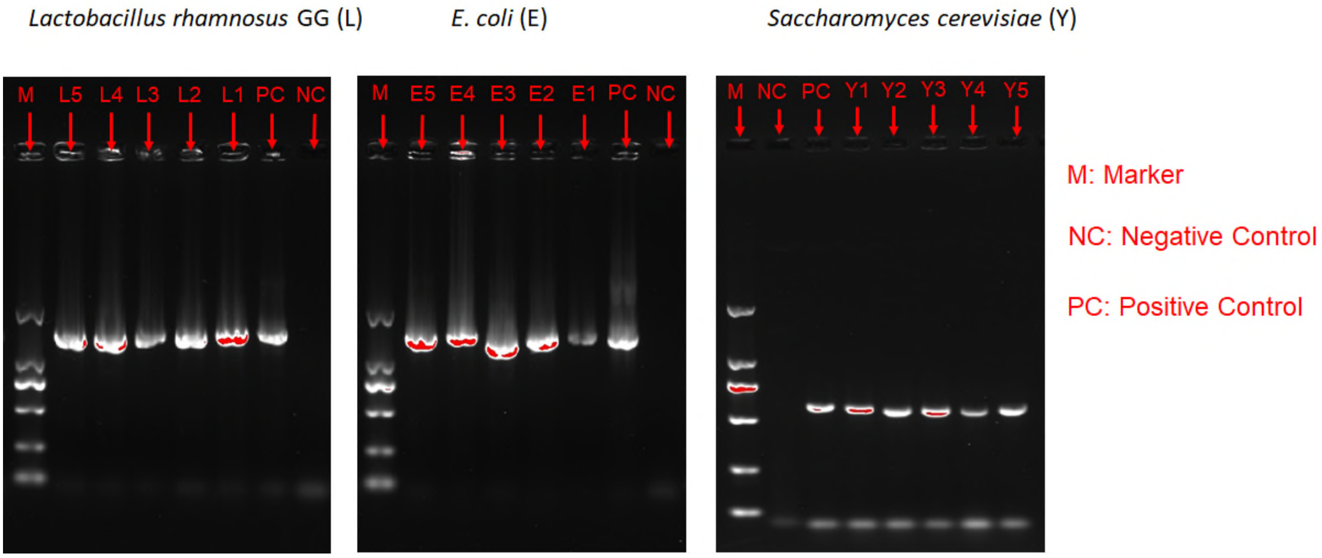

Supplementary Figure S3. PCR results from culturable colonies after single cell ejection. The picture was taken by ChemiDoc™ MP Imaging System (Bio-Rad)

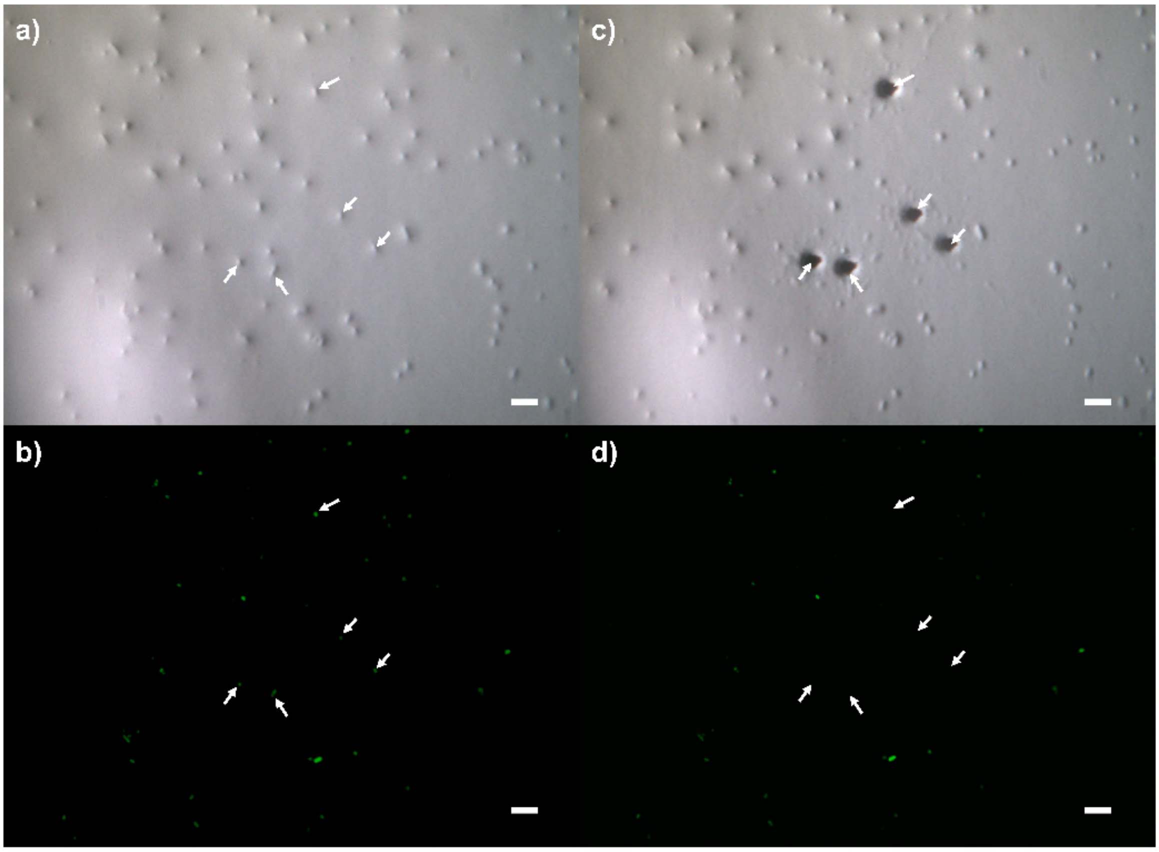

Supplementary Figure S4. Fluorescence isolating single *E. coli* JM109 cells with GFP plasmid from *E. coli* DH5α by LIFT. a) Image before ejecting. b) Fluorescence image before ejecting. c) Image after ejecting. b) Fluorescence image after ejecting. The bar represents 10μm.

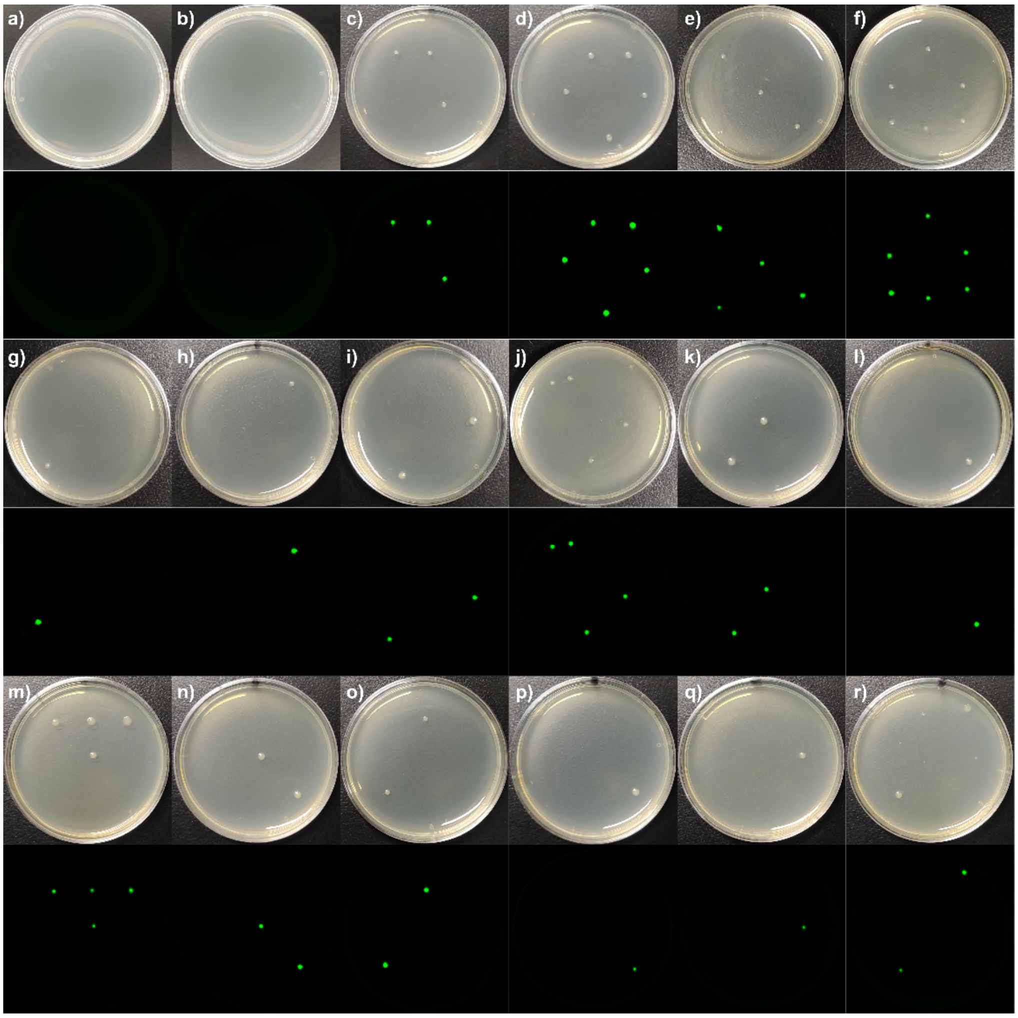

Supplementary Figure S5. Culturing results of isolated single Jm109 cells from DH5α, cultured after 24h, the black picture below each panel is the corresponding fluorescence picture, taken by ChemiDoc MP Imaging System (BIO-RAD) operating on DyLight 488 mode (at 488 and at 532/28).

Panel a: control group which was placed in the air. Panel b: control group which ejecting the blank place around the cell and receiving. Panels c-i show the first experiment, we ejected 9 single cells in each petri dish and 81 single cells on 9 petri dishes totally, about 22 colonies grow on 7 petri dishes. Panels j-r show the second experiment, we ejected 9 single cells in each petri dish and 81 single cells on 9 petri dishes totally, about 19 colonies grow on 9 petri dishes. The single cell's recultivation ratio is about 25.3% (41/162).

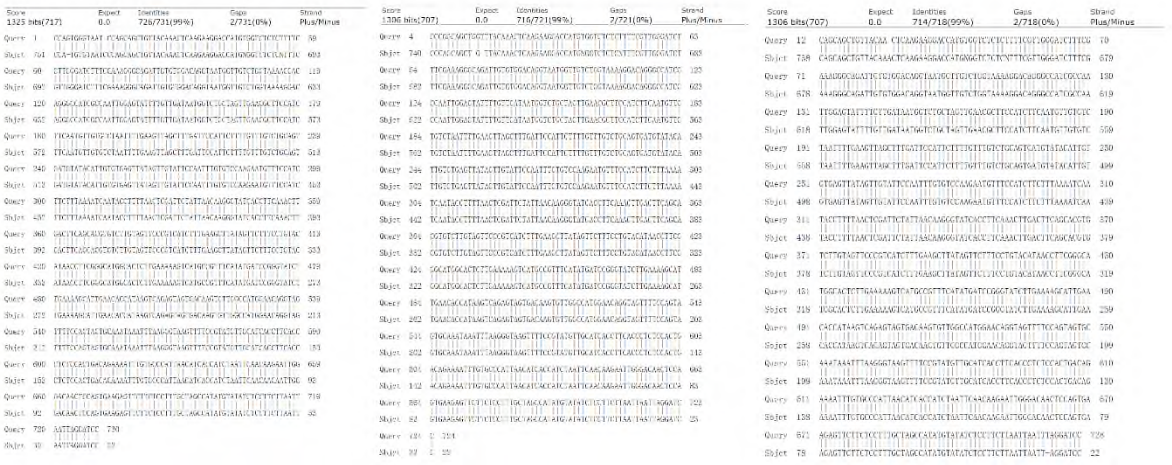

Supplementary Figure S6. Plasmid sequencing results of the recultivated *E. coli* JM109 (pGFP).s

SPARKeasy Superpure Mini Plasmid Kit (SparkJade, AD0102-B) was used for plasmid extraction.

(1) Pick and transfer 5 colonies on the sorting culture plate to a 3ml culture tube, and culture overnight at 37°C and 200rpm shaking;

(2) Centrifuge the 3ml bacterial solution cultured overnight, centrifuge at 13000g for 1min, and collect the bacterial sediment;

(3) Add 250μl of solution P1 to resuspend the bacterial pellet, shake and mix well;

(4) Add 250μl of solution P2, gently turn up and down and mix 3-5 times, and leave it at room temperature for 4 minutes;

(5) Add 350μl of solution P3, immediately and gently mix up and down for 7-8 times to produce a white flocculent precipitate;

(6) Centrifuge at 13000g for 10 min, and collect the supernatant;

(7) Add the supernatant to the adsorption column AC, put the adsorption column into the collection tube, centrifuge at 13000g for 1 min, and discard the waste liquid.

(8) Add 500μl of deproteinized liquid PE, centrifuge at 13000g for 1min, discard the waste liquid;

(9) Add 600μl rinsing solution WB, centrifuge at 13000g for 1min, discard the waste liquid;

(10) Repeat the previous step;

(11) Put the AC column into the empty collection tube, centrifuge at 13000g for 2 minutes to remove the WB residual liquid;

(12) Remove the AC column and put it in a new centrifuge tube, add 30-70μl of eluent EB in the middle of the adsorption membrane, leave it at room temperature for 2 minutes, and centrifuge at 13000g for 1 minute;

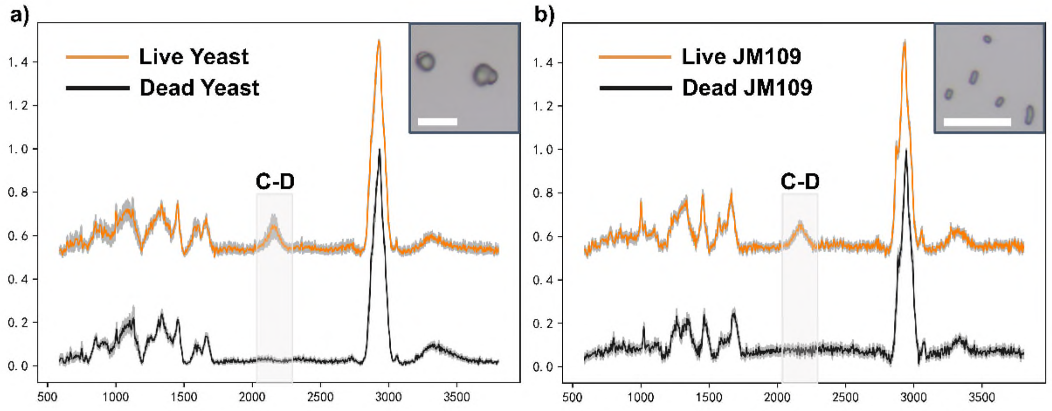

Supplementary Figure S7. a) Raman spectroscopy of recultivated yeast. b) Raman spectroscopy of recultivated JM109. The bar represents 10 μm.

The two experiment groups were handled for Raman spectroscopy, as shown in figure, C-D band (2040–2300 cm−1) exists could prove that the cell's active metabolism.

a

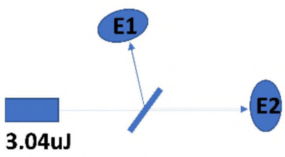

Thickness of AL	E1	E2	Absorption (%)
25nm(Domestic)	2.33uJ	124.6nJ	19.26
25nm(Imported)	2.50uJ	51.4nJ	16.07
35nm	2.53uJ	23.0nJ	16.02
45nm(Imported)	2.65uJ	7.8nJ	12.57
45nm(Domestic)	2.53uJ	8.0nJ	16.51
60nm(Imported)	2.60uJ	0	14.47
60nm(Domestic)	2.60uJ	0	14.47
80nm	2.67uJ	0	12.17
100nm(Imported)	2.70uJ	0	11.18
100nm(Domestic)	2.58uJ	0	15.13
150nm	2.71uJ	0	10.86
200nm	2.68uJ	0	11.84

b

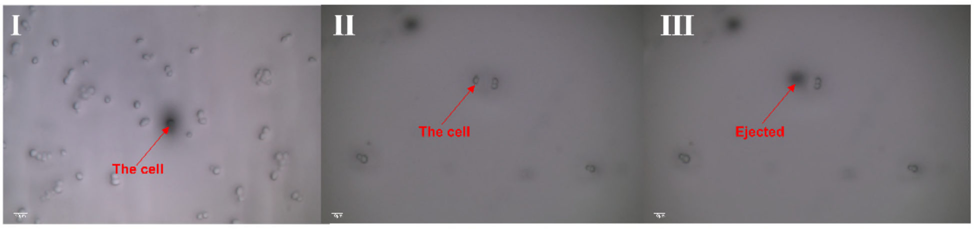

Supplementary Figure S8 (a): The absorption of different thickness of aluminum film;

(b): ejection of three-layer LIFT. I: ejection with inadequate energy, the agar film didn't break and the cell were not ejected successfully, II: two cells were close, III: with a suitable ejecting energy, only one cell was ejected with no broken of agar film.

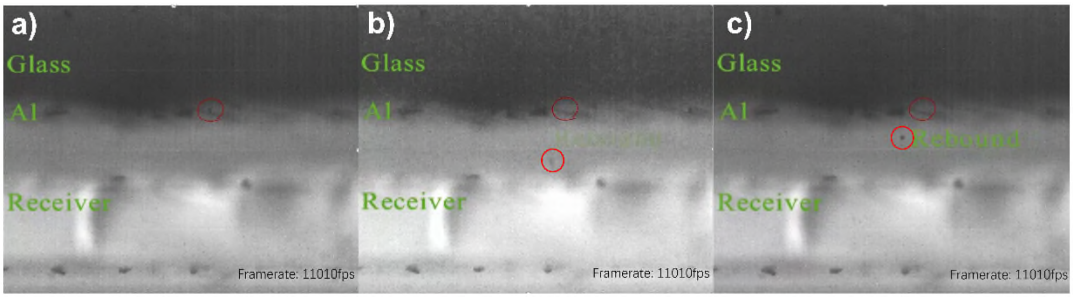

Supplementary Figure S9. a) The yeast cell on the LIFT chip; b) The ejected yeast cell hit the receiver (cover glass); c) The yeast cell rebound to highest position and fall slowly again.

Detail could be seen in supplementary video 1

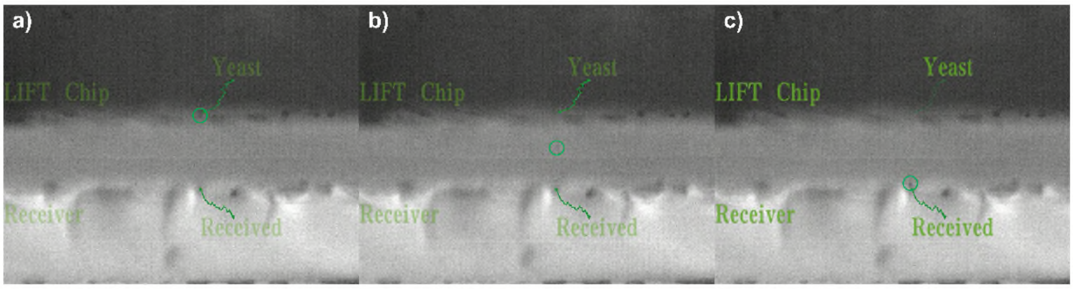

Supplementary Figure S10. Flying and landing processes of single yeast cell. a): the yeast cell on the LIFT chip; b): the yeast cell is flying in the air between the LIFT chip and receiver; c): the yeast cell lands on the receiver. The velocity could be calculated about 0.35m/s, and the whole process was shown in supplementary video 2.

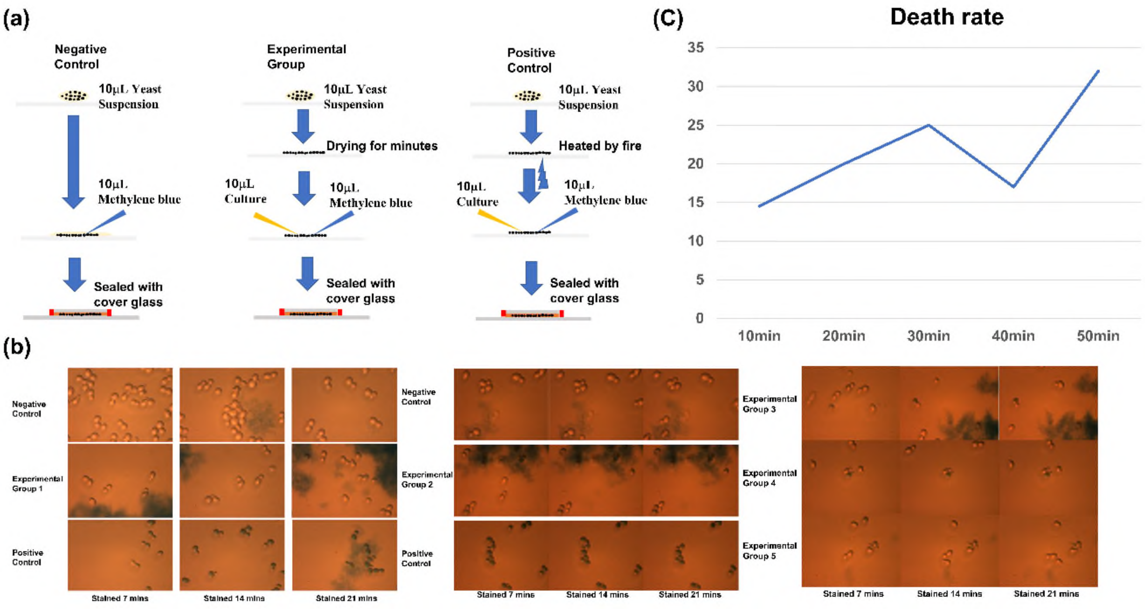

Supplementary Figure S11. The death rate of yeast cell drying with time on LIFT chip.

a): diagram of the experiment setup: three groups (Negative group: 10 μL yeast suspension stained with 10 μL 200mg/L Methylene blue; Experimental group: 10 μL yeast suspension drying for 10, 20, 30, 40, 50 minutes respectively, stained with 10 μL culture and 10 μL 200mg/L Methylene blue; Positive group: 10 μL yeast suspension heated with fire for 20 seconds, stained with 10 μL culture and 10 μL 200mg/L Methylene blue;) of yeast cells were stained on LIFT chip, sealed with cover glass and recorded under microscope.

b): picture of each group of cells after stained 7, 14, 21 minutes.

c): death rate curve of yeast cell with drying time (count according to video recorded)

Supplementary Table S1. Physical parameters of materials

Physical parameters of materials	Aluminum	Agar (w = 0.1)	Water
ρ: density (kg/m^3)	2700	1065	1000
k: thermal conductivity ($W/m.K$)	238	0.566	0.599
C_p: heat capacity ($J/kg.K$)	900	4200	4200

Supplementary Table S2. Statistics of *S. cerevisiae*, *E. coli* and LGG's culturing results

**Number of ejected cells in each microwell**		**Replicate 1**	**Replicate 2**	**Replicate 3**	**Replicate 4**	**Replicate 5**	**Recovery rate**
S. cerevisiae	**1**	3	5	5	7	6	
	**1**	5	6	6	7	7	63%
E. coli	**1**	4	1	1	2	1	
	**1**	5	1	2	2	1	22%
***L. rhamnosus* GG (LGG)**	**1**	6	5	2	5	5	
	**1**	9	9	8	9	9	74%
